# Mitochondrial Dysfunction and Apoptosis in Cumulus Cells of Type I Diabetic Mice

**DOI:** 10.1371/journal.pone.0015901

**Published:** 2010-12-28

**Authors:** Qiang Wang, Antonina I. Frolova, Scott Purcell, Katie Adastra, Erica Schoeller, Maggie M. Chi, Tim Schedl, Kelle H. Moley

**Affiliations:** 1 Department of Obstetrics and Gynecology, Washington University School of Medicine, St. Louis, Missouri, United States of America; 2 Department of Genetics, Washington University School of Medicine, St. Louis, Missouri, United States of America; Cincinnati Children's Research Foundation, United States of America

## Abstract

Impaired oocyte quality has been demonstrated in diabetic mice; however, the potential pathways by which maternal diabetes exerts its effects on the oocyte are poorly understood. Cumulus cells are in direct contact with the oocyte via gap junctions and provide essential nutrients to support oocyte development. In this study, we investigated the effects of maternal diabetes on the mitochondrial status in cumulus cells. We found an increased frequency of fragmented mitochondria, a decreased transmembrane potential and an aggregated distribution of mitochondria in cumulus cells from diabetic mice. Furthermore, while mitochondrial biogenesis in cumulus cells was induced by maternal diabetes, their metabolic function was disrupted as evidenced by lower ATP and citrate levels. Moreover, we present evidence suggesting that the mitochondrial impairments induced by maternal diabetes, at least in part, lead to cumulus cell apoptosis through the release of cytochrome *c*. Together the deleterious effects on cumulus cells may disrupt trophic and signaling interactions with the oocyte, contributing to oocyte incompetence and thus poor pregnancy outcomes in diabetic females.

## Introduction

In women, type I (insulin-dependent) diabetes has been linked to complications during pregnancy, often resulting in miscarriage, embryonic developmental abnormalities and congenital malformations [1]. Likewise, numerous studies have suggested that the diabetic condition adversely affects development of pre- and post-implantation embryos in rodents [2], [3], [4], [5]. Recently, emerging evidence has shown that oocytes from diabetic mice experience delayed maturation, abnormal cellular metabolism, mitochondrial dysfunction and meiotic defects [6], [7], [8]. These changes in the oocyte may be manifested later as developmental abnormalities in preimplantation embryos, congenital malformations, and even metabolic disease in the offspring [8], [9]. However, the pathway(s) by which maternal diabetes exerts its effects on the oocyte remains ill defined.

Mammalian ovarian follicles are highly specialized structures that support the growth and development of oocytes. Bidirectional communication between the oocyte and companion somatic cells, known as the granulosa cells, is essential for the development and function of both follicular compartments [10]. In antral follicles, there are two major types of granulosa cells that are anatomically and functionally distinct: mural granulosa cells, which line the wall of the follicle and play a principally steroidogenic role, and cumulus cells, which form an intimate association with the oocyte. Cumulus cells possess specialized trans-zonal cytoplasmic projections that penetrate through the zona pellucida and form gap junctions at their tips with the oocyte, generating an elaborate structure called the cumulus-oocyte complex (COC) [11], [12]. Cumulus cells have long been known to play a nurturing role in supporting oocyte development by providing essential nutrients to oocytes [13]. Recently, we detected abnormal metabolism, increased apoptosis and decreased gap junction communication in granulosa cells from diabetic mice [8], [14], [15], [16]. Moreover, mitochondria are the primary energy-generating system in most eukaryotic cells, participating in intermediary metabolism and apoptosis.

Given the above findings, we hypothesized that maternal diabetes adversely impacts the mitochondria in cumulus cells, which may be further transferred into the oocyte, contributing to poor oocyte quality. To test this hypothesis, we investigated the effects of maternal diabetes on mitochondrial status in cumulus cells using streptozotocin (STZ)-induced diabetic and Akita (insulin 2 gene mutant) mouse models. Molecular, cellular and biochemical analysis demonstrated structural, spatial and metabolic dysfunction of mitochondria in cumulus cells of diabetic mice. Furthermore, we provide evidence that mitochondrial impairments are involved in apoptosis of cumulus cells induced by maternal diabetes.

## Results

### Morphological alterations of mitochondria in cumulus cells of diabetic mice

**Figure 1:**
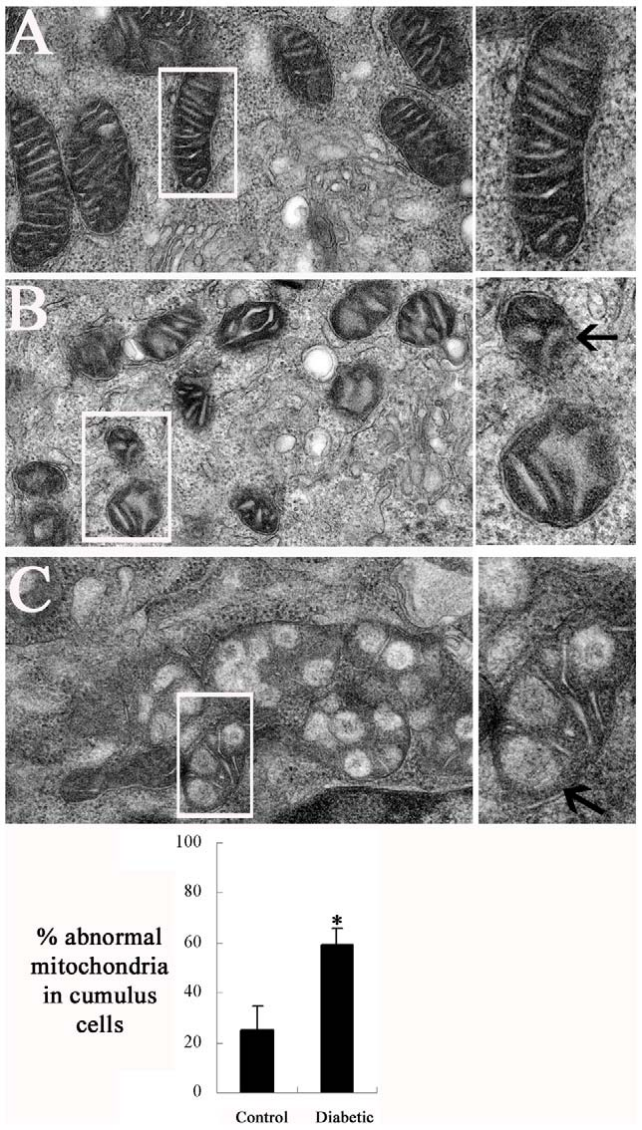
Altered mitochondrial morphology in cumulus cells of diabetic mice. Cumulus-oocyte complexes collected from control and diabetic mice were subjected to transmission electron microscopy analysis and mitochondrial structure was compared. (A) Representative electron micrograph of mitochondria from control cumulus cells, showing bean-shaped structures with numerous transversely orientated cristae enveloped by an intact outer membrane; (B–C) Representative electron micrographs show alterations in mitochondrial morphology of diabetic cumulus cells: (B) small spherical mitochondria with fewer and disarrayed cristae and a decreased electron density of the matrix (arrow), and (C) mitochondria with membrane rupture or large vacuoles (arrow). Higher magnification views of boxed regions are also presented (right panel). (D) Quantification analyses of abnormal mitochondria in cumulus cells from control and diabetic mice. Data are presented as mean percentage of abnormal mitochondria ± SD in total examined mitochondria. * p<0.05 vs control.

To determine if maternal diabetes affects mitochondrial structure in cumulus cells, transmission electron microscopy (TEM) was performed on cumulus-oocyte complexes (COCs) from control and diabetic mice. Representative photomicrographs are shown in Fig. 1. Most mitochondria in the control cumulus cells presented as bean-shaped structures with numerous transversely orientated cristae enveloped by an intact outer membrane (Fig. 1A). In striking contrast, we observed a higher frequency of morphological alterations in cumulus cell mitochondria from diabetic mice (60±7% vs 25±9% control, p<0.05; Fig. 1D). They displayed small spherical structures with fewer and disarrayed cristae, and a decreased electron density of the matrix (Fig. 1B, arrow). In addition, an increased proportion of membrane rupture and the presence of vacuoles in mitochondria (Fig. 1C, arrow) were found in diabetic cumulus cells as compared to controls. These ultrastructural defects have been correlated with mitochondrial fission, metabolic disorders and cell death [17], [18].

### Decrease in mitochondrial membrane potential in cumulus cells of diabetic mice

**Figure 2:**
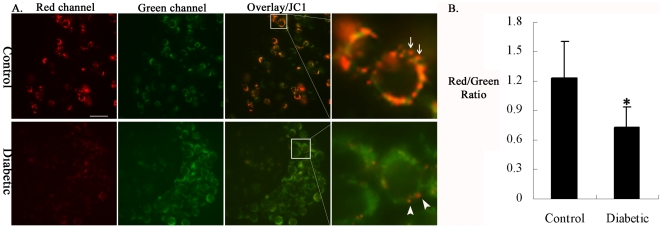
Decreased mitochondrial membrane potential in cumulus cells of diabetic mice. Cumulus-oocyte complexes from control and diabetic mice were stained with JC-1 to evaluate mitochondrial membrane potential (Δψ_m_) by fluorescence microscopy. Representative images of cumulus cells are shown. (A) Generally, mitochondria in cumulus cells of control mice were predominantly in red form (arrows), indicating the high Δψ_m_. In contrast, the loss of red fluorescence and thus increased green mitochondria were observed in cumulus cells of diabetic mice (arrowheads), indicating the low Δψ_m_. (B) Histogram shows the ratio of red to green fluorescence intensity calculated to characterize Δψ_m_. Note the decreased Δψ_m_ in cumulus cells of diabetic mice. Error bars indicate ± SD. * p<0.05 vs controls. Scale bar: 20 µm.

In light of defects in mitochondrial structure described above, we assessed whether there was any alteration in mitochondrial membrane potential (Δψ_m_) in cumulus cells of diabetic mice. COCs from control and diabetic mice were stained with JC-1, a fluorescent probe that selectively enters mitochondria and reversibly changes color from green to red as the Δψ_m_ increases [19], and then examined by fluorescence microscopy. As shown in Fig. 2A, mitochondria in control and diabetic cumulus cells were a combination of both low and high membrane potential organelles, as evident by the green and the red fluorescence. In general, mitochondria in the control cells had predominantly high membrane potential as indicated by the red fluorescence (Fig. 2; arrows). However, a trend towards lower membrane potential, loss of red fluorescence and accordingly increased green mitochondria, was observed in the diabetic cumulus cells (Fig. 2A; arrowheads). For quantitative analysis, we measured the intensity of red and green fluorescence, and then the ratios of red/green were calculated to characterize Δψ_m_. Notably, the red/green ratio was significantly decreased in the cumulus cells of diabetic mice as compared to those of control mice (0.73±0.20 vs 1.23±0.38 control, p<0.05; Fig. 2B). Such a fluorescence shift from red to green suggests a general drop of mitochondrial membrane potential in cumulus cells exposed to maternal diabetes.

### Mitochondrial redistribution in cumulus cells of diabetic mice

**Figure 3:**
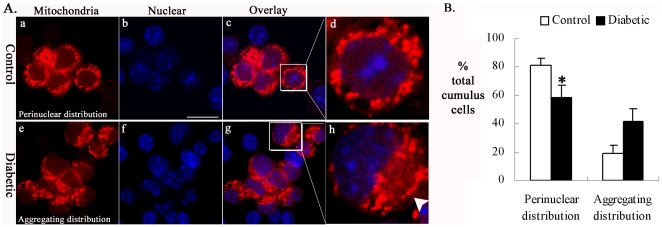
Mitochondrial distribution was disrupted in cumulus cells of diabetic mice. Cumulus-oocyte complexes collected from control and diabetic mice were labeled with MitoTracker Red to visualize mitochondrial localization and costained with DAPI to visualize nuclei. Representative confocal sections of cumulus cells are shown. (A) Mitochondria of most cumulus cells from control mice show perinuclear distribution pattern (a–d). Notably, in cumulus cells from diabetic mice, aggregating distribution pattern of mitochondria was readily observed (e–h; arrowhead). (B) Quantification of cumulus cells with each mitochondrial distribution pattern from control and diabetic mice. Data are expressed as mean percentage ± SD from three independent experiments in which at least 200 cells were analyzed. * p<0.05 vs control. Scale bar: 20 µm.

Trafficking of mitochondria is thought to be important for mammalian cells to be able to cater to differing energy requirements and provide a means for environmental sensing [20]. To determine whether maternal diabetes affects the spatial organization of mitochondria in cumulus cells, we compared the mitochondrial localization between cumulus cells from control and diabetic mice. Cells were examined by confocal microscopy following MitoTracker staining. Roughly, mitochondrial distribution patterns in the cumulus cells could be classified into two different categories: (1) mitochondria surrounding the nucleus, termed perinuclear distribution (Fig. 3Aa–d), and (2) mitochondria aggregated in parts of the cytoplasm, termed aggregating distribution (Fig. 3Ae–h; arrowhead). By performing quantitative analysis, we found that the percentage of the perinuclear distribution pattern was significantly decreased in diabetic cumulus cells relative to control (58±9% vs 81±5% control, p<0.05; Fig. 3B), whereas the proportion of the aggregating distribution pattern was concomitantly increased relative to control (42±9% vs 19±5% control, p<0.05; Fig. 3B). These results indicate a redistribution of mitochondria in cumulus cells from diabetic mice.

### Increased mitochondrial biogenesis in cumulus cells of diabetic mice

**Figure 4:**
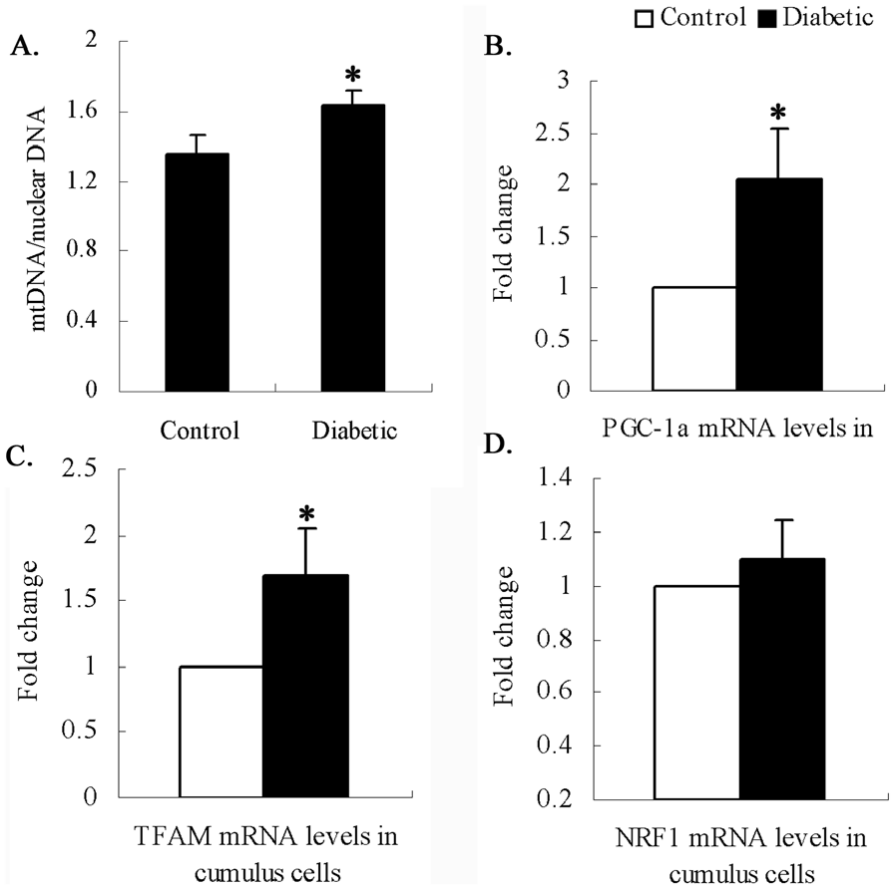
Increased mitochondrial biogenesis in cumulus cells of diabetic mice. Cumulus cells removed from cumulus-oocyte complexes were collected for analysis. (A) mtDNA content was calculated using quantitative real-time PCR by measuring the ratio of cytochrome b (mitochondrial gene) to β-actin (nuclear gene) DNA levels in cumulus cells of control and diabetic mice. (B–D) mRNA levels of genes implicated in mitochondrial biogenesis determined by real-time RT-PCR in cumulus cells from control and diabetic mice. At least three experiments were performed and data are presented as the mean ± SD of the fold changes. * p<0.05 vs control.

To investigate the potential effects of maternal diabetes on mitochondrial biogenesis in cumulus cells, we first evaluated mitochondrial DNA (mtDNA) content in cumulus cells from control and diabetic mice by quantitative real-time PCR. Data are expressed as the ratio of mtDNA to nuclear DNA, as shown in Fig. 4A. Surprisingly, we found that mtDNA content in cumulus cells was significantly higher in diabetic mice than in control mice (1.63±0.09 vs 1.35±0.12 control; p<0.05). Further, we measured the mRNA levels of genes implicated in mitochondrial biogenesis, such as peroxisome proliferator-activated receptor gamma coactivator 1 alpha (PGC-1α), nuclear respiratory factor 1 (NRF1) and mitochondrial transcription factor A (TFAM) [21]. In agreement with elevated mtDNA content, the cumulus cells of diabetic mice demonstrated an approximate 2-fold increase in PGC-1αand TFAM mRNA expression compared with those of control mice (Fig. 4B and C). No significant difference was detected for NRF1 transcripts (Fig. 4D). Together these data demonstrate a mitochondrial biogenic response in cumulus cells of diabetic mice.

### Metabolic dysfunction of mitochondria in cumulus cells of diabetic mice

**Figure 5:**
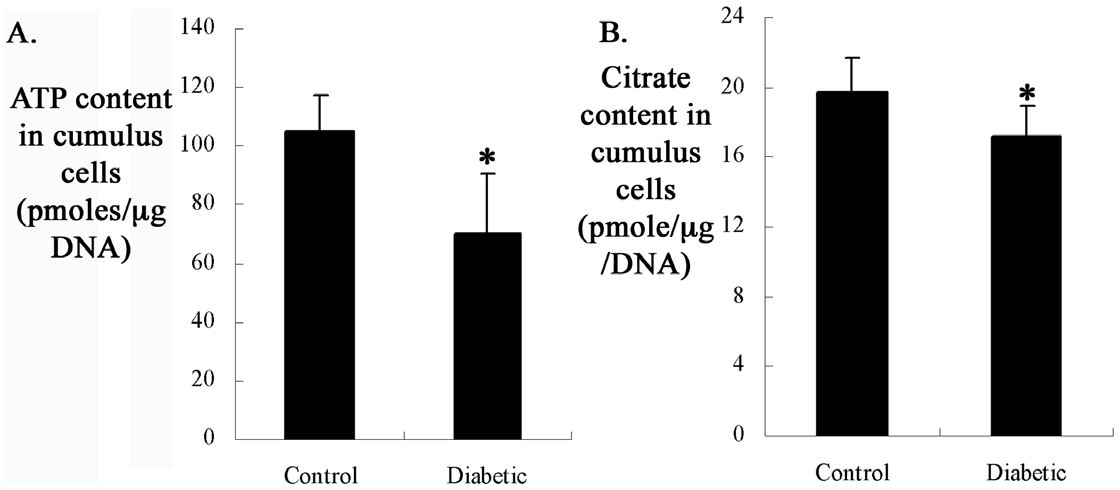
Reduced ATP and citrate content in cumulus cells of diabetic mice. Cumulus cells removed from cumulus-oocyte complexes were collected to determine the levels of ATP and citrate. Values are expressed as pmoles per µg DNA. (A–B) Histogram shows the average ATP and citrate content in cumulus cells from control and diabetic mice. All measurements were performed in triplicate. Error bars indicate ± SD. * p<0.05 vs controls.

Given the alterations in mitochondrial structure and biogenesis, we asked whether mitochondrial metabolism was disrupted in cumulus cells of diabetic mice. Mitochondria generate most of cell's supply of ATP, and the only source of citrate in the cell is mitochondrial tricarboxylic acid cycle (TCA) [22]. Hence, to address the question above, cumulus cells separated from COCs of control and diabetic mice were processed to measure ATP and citrate levels. All metabolites are expressed as pmoles/µg DNA (see Methods). As shown in Fig. 5, the average ATP and citrate levels were both markedly reduced in the cumulus cells of diabetic mice as compared to controls (ATP: 70.3±19.8 vs 105.0±12.2 control, p<0.05; Citrate: 17.2±1.7 vs 19.8±2.1 control, p<0.05), suggesting a decline of mitochondrial function.

### Mitochondrial/cytochrome *c* pathway is involved in apoptosis in cumulus cells of diabetic mice

**Figure 6:**
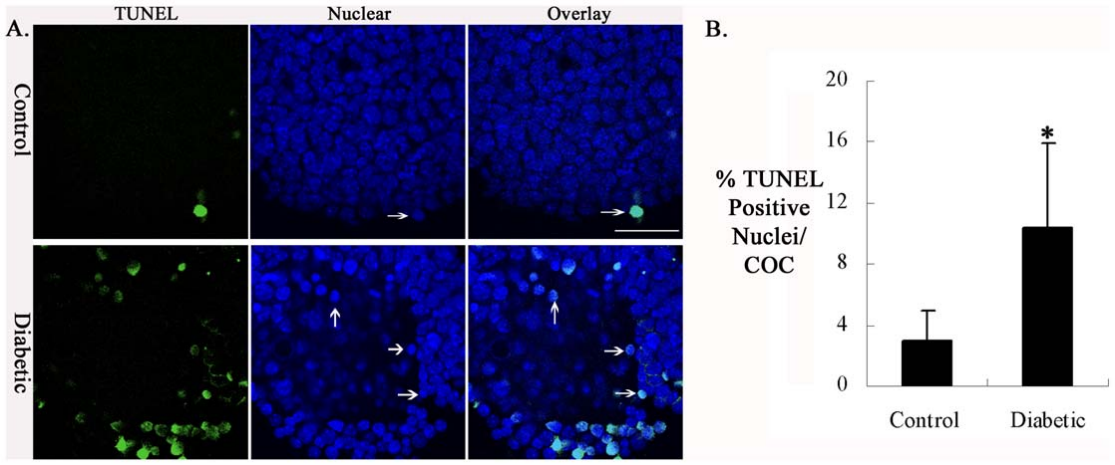
Increased apoptosis in cumulus cells of diabetic mice. (A) Cumulus-oocyte complexes from control and diabetic mice were stained with TUNEL to visualize apoptotic cells (green) and counterstained with DAPI to confirm nuclear status (blue). Arrows indicate the condensed nuclei of apoptotic cells. Representative confocal sections of cumulus cells are shown. (B) Frequency of TUNEL-positive nuclei in cumulus cells from control and diabetic mice. Data represent mean ± SD of three independent experiments in which at least 30 COCs were analyzed. * p<0.05 vs controls. Scale bar: 20 µm.

**Figure 7:**
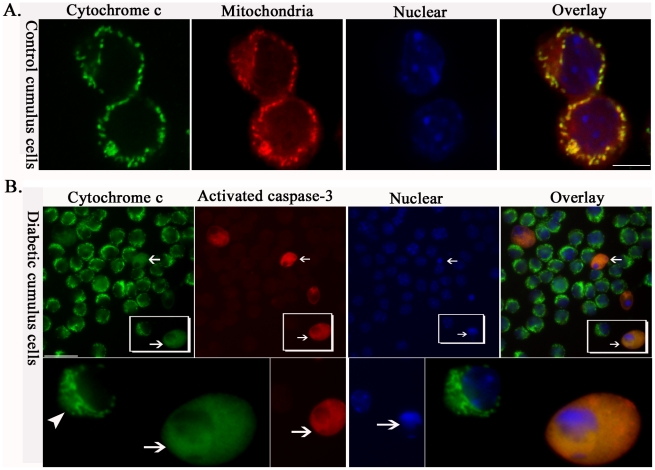
Cytochrome *c* translocation and caspase-3 activation in apoptotic cumulus cells of diabetic mice. (A) Cumulus-oocyte complexes collected from control mice were stained with cytochrome c antibody and MitoTracker to determine the subcellular localization of cytochrome c (green) and mitochondria (red), and counterstained with DAPI to visualize nuclei (blue). Representative confocal sections of cumulus cells are shown. Cytochrome *c* shows a punctuate distribution pattern and co-localizes with the mitochondria (yellow) in control cumulus cells. (B) Cumulus-oocyte complexes collected from diabetic mice were stained with cytochrome c antibody (green), activated caspase-3 antibody (red) and DAPI (blue). All apoptotic cumulus cells of diabetic mice (arrows), as evidenced by positive staining of active caspase-3 antibody (red; arrows) and condensed chromatin (blue; arrows), always display diffuse staining of cytochrome *c* (green; arrows), indicating its translocation from mitochondria to cytoplasm. In contrast, those non-apoptotic cumulus cells of diabetic mice (green, lower panel; arrowheads) stained negatively with active caspase-3 antibody still show mitochondria-localized cytochrome *c*. Scale bars: 20 µm.

In mammalian cells, signaling cascades leading to apoptosis can be divided into two broad groups. The intrinsic pathway (also called mitochondrial pathway) is characterized by the central role of mitochondria in the initiation of the caspase cascade executing the apoptotic program. In the extrinsic pathway, caspase activation is triggered by death receptors on the cell surface [23]. As described above (Fig. 1 and 2), we have detected altered mitochondrial morphology and reduced Δψ_m_ in the cumulus cells of diabetic mice. These abnormalities have been widely reported as being sufficient to activate the apoptotic program by promoting cytochrome *c* release from mitochondria into the cytoplasm [24], [25]. Thus, one possibility is that the observed mitochondrial defects lead to cumulus cell apoptosis in diabetic mice through cytochrome *c* translocation. To address this possibility, we first evaluated apoptosis in cumulus cells from control and diabetic mice using the TUNEL assay coupled with confocal microscopy (Fig. 6A). Condensed chromatin (Fig. 6A; arrows) can be observed in apoptotic cumulus cells, indicated by positive TUNEL staining. Quantitative analysis demonstrated a significant increase in the incidence of cumulus cell apoptosis from diabetic mice as compared with controls (10.4±5.6% vs 3.0±1.9% control; Fig. 6B). We next examined whether the subcellular localization of cytochrome *c* was altered in those apoptotic cumulus cells from diabetic mice using immunostaining [26]. Confocal microscopy clearly revealed a punctate distribution pattern of cytochrome *c* in control cumulus cells, which co-localized with the mitochondria-specific dye, MitoTracker Red (Fig. 7A). However, apoptotic cumulus cells from diabetic mice (Fig. 7B; arrows), as evidenced by positive staining with the active caspase-3 antibody (red) and condensed chromatin (blue) [27], always displayed a diffuse staining of cytochrome *c* in the cytoplasm (green). This observation suggests that there is cytochrome *c* loss from mitochondria/translocation to the cytoplasm. Those non-apoptotic cumulus cells of diabetic mice, which are stained negatively with the active caspase-3 antibody, retained mitochondria-localized cytochrome *c*, similar to control cells (Fig. 7B; arrowhead, lower panel). Taken together, the co-occurrence of cytochrome *c* release, casapase-3 activation and apoptosis suggest that maternal diabetes induced-apoptosis in cumulus cells is mediated, at least in part, by the mitochondrial pathway.

Facilitative glucose transporters (GLUTs) are essential for the glucose transport activity in cells. Glucose limitation related with GLUT1 deficiency has been reported to result in a decrease in mitochondrial membrane potential, cytochrome *c* redistribution to cytosol, and subsequent activation of mitochondria-dependent apoptosis [28], [29]. We therefore tentatively examined GLUT1 expression and glucose uptake (File S1). We detected a dramatic downregulation of GLUT1 protein expression (Fig. S1) and concomitant glucose uptake (Fig. S2) in diabetic cumulus cells compared with controls. It is therefore possible that glucose deprivation may trigger the mitochondrial impairments and apoptosis in diabetic cumulus cells. Regardless, the exact mechanisms underlying this process remain to be uncovered.

### Analysis of cumulus cells from Akita genetic diabetic model

Akita mouse, a diabetic model with spontaneous mutation of insulin 2 gene [30], was used to test whether the abnormalities in cumulus cells were caused by streptozotocin itself rather than maternal diabetes. Some key phenotypes were checked and similar results were obtained. Mitochondrial membrane potential was dramatically reduced in cumulus cells from Akita mice in comparison with wild type mice (Fig. S3A–B). Confocal microscopy revealed a significantly higher percentage of apoptosis in Akita cumulus cells than in WT (16.8±5.2% vs 7.7±4.7% WT; Fig. S4A–B). Compared to WT, Akita mice also demonstrated the decreased GLUT1 expression (Fig. S1B) and glucose uptake (File S1; 1.85±0.53 vs 2.49±0.46 counts/µg protein WT; Fig. S2B) in cumulus cells.

## Discussion

In the present study, we revealed alterations in morphology, distribution, biogenesis and metabolism of mitochondria in cumulus cells of diabetic mice, suggesting mitochondrial dysfunction. Furthermore, cumulus cells in diabetic mice undergo apoptosis at increased frequency, likely via the mitochondrial, cell intrinsic, pathway.

### Effects of maternal diabetes on the mitochondrial status in cumulus cells

Mitochondria are dynamic organelles, and their length, shape and size are controlled by precisely regulated rates of fusion and fission [31]. An imbalance of these two processes can dramatically alter the overall mitochondrial morphology [17]. Using TEM, we observed a high frequency of mitochondrial morphological anomalies in cumulus cells of diabetic mice, displaying small spherical structures with fewer and disarrayed cristae (Fig. 1)—these are often referred to as “fragmented mitochondria” [18]. This suggests that the maternal diabetic condition disrupts mitochondrial dynamics in cumulus cells, resulting in greater mitochondrial fission. Mounting evidence suggests that fusion and fission of mitochondria affects the ability of cells to distribute their mitochondria to specific subcellular location. For example, mitofusins (MFN1 and MFN2) are dynamin family GTPases known to be essential for mitochondrial fusion [32]. Aggregation of mitochondria has been observed in cell lines expressing MFN mutants [32], [33] and MFN2-deficient neurons and myotubes [34], [35]. Remarkably, our immunofluorescent data revealed that maternal diabetes leads to a mitochondrial redistribution in cumulus cells. Increased mitochondrial aggregates were readily observed in diabetic cumulus cells as compared to controls (Fig. 3). The mechanism of such mitochondrial clusters is hypothesized to be attributable to the formation of tethered intermediates, which are unable to complete mitochondrial fusion, whereby ongoing fission then leads to fragmented-tethered mitochondrial clusters [35], [36]. Furthermore, it is generally thought that trafficking of mitochondria is important for cellular function by placing them in appropriate locations relative to energy requiring process [20]. Hence, those fragmented and mislocated mitochondria in the cumulus cells of diabetic mice may result in subcellular energy depletion and even cell death [23], [37], [38].

Mitochondrial biogenesis is a complex process involving the coordinate expression of mitochondrial and nuclear genes, import of products of the latter into the organelle and turnover [39]. It has been documented that PGC-1αregulates mitochondrial biogenesis by serving as a coactivator of multiple transcription factors, such as NRF and TFAM [40], [41], [42]. Our quantitative real-time PCR analysis showed that mtDNA content was significantly increased in cumulus cells from diabetic mice when compared to those from controls. In agreement, PGC-1α and TFAM mRNAs were upregulated in the diabetic cumulus cells (Fig. 4). To our surprise, although mitochondrial biogenesis is stimulated, their metabolic function was reduced in the cumulus cells of diabetic mice (Fig. 5). Increased mtDNA content and impaired mitochondrial function have also been reported in several other diabetic and aged tissues [43], [44], [45], [46], [47]. Taking into account the decreased GLUT1 expression and glucose uptake in diabetic cumulus cells (Fig. S1 and S2), we speculate that the increased mitochondrial biogenesis may be a compensatory response to impaired glycolytic metabolism and dysfunctional mitochondria in cumulus cells of diabetic mice.

It is known that mitochondria play diverse roles in cell physiology and pathology including regulation of apoptosis, participation in ion homeostasis and transport of metabolites. These important functions are highly dependent on the transmembrane potential [48]. By JC-1 staining, we found that Δψ_m_ was markedly reduced in the cumulus cells of diabetic mice as compared to control cells (Fig. 2). On the one hand, Δψ_m_ has been demonstrated to be essential for mitochondrial fusion, although the mechanistic link between them remains to be resolved. Ionophores that dissipate the mitochondrial membrane potential cause mitochondrial fragmentation, owing to an inhibition of mitochondrial fusion [49], [50]. On the other hand, loss of Δψ_m_ could induce the opening of the permeability transition pore in the inner mitochondrial membrane and consequent rupture of the outer mitochondrial membrane, which may allow the release of proteins that reside in the intermembrane space, including cytochrome *c*, to activate the caspase cascade that executes the apoptotic program [51], [52]. In line with this notion, we found that cytochrome *c* is released from the mitochondria to the cytoplasm in all apoptotic cumulus cells of diabetic mice, coinciding with activation of caspase-3 (Fig. 6 and 7), implicating the mitochondrial death pathway in maternal diabetes-induced cumulus cell apoptosis. In addition, the potential role of mitochondrial fragmentation and redistribution, which we observed in cumulus cells from diabetic mice, has also been described as participating in the control of apoptosis [25], [53], [54].

Collectively, our data demonstrates that maternal diabetes induces mitochondrial dysfunction in cumulus cells, which leads, at least in part, to an increase in apoptosis, probably by regulating GLUT1 expression and concomitant glucose transport.

### Potential effects of mitochondrial dysfunction and apoptosis in cumulus cells on oocyte quality in diabetic mice

Cumulus cells and the oocyte are metabolically coupled throughout follicular development by membrane specializations know as gap junctions [55]. It is well known that cumulus cells support oocyte development through the provision of essential nutrients, information molecules, metabolic precursors and signaling molecules [56]. An important point, particularly in relation to diabetes, is that oocytes are deficient in their ability to use glucose as an energy substrate and require cumulus cell-provided products of glycolysis like pyruvate for their own development [55]. For example, only pyruvate can support oocyte development in vitro if no cumulus cells are present. However, when these cells are included in the culture medium glucose is also able to support oocyte maturation [57]. Notably, pyruvate can easily move into the mitochondrial matrix which contains pyruvate dehydrogenase, where it enters TCA cycle generating energy such as ATP [58]. Moreover, oocytes enclosed in cumulus cells have higher amounts of ATP than those lacking cumulus cells [59], suggesting that cumulus cells provide ATP for oocyte development. Pyruvate produced as a product of glycolysis by cumulus cells also could be transferred to oocytes via gap junction [10]. Thus, mitochondrial function is critical for the energy production through glycolysis and TCA cycle pathways in cumulus cells and thereafter energy supply for oocyte maturation.

Combining our previous findings [14], [16] with the results presented here, we conclude that mitochondrial dysfunction and the resultant apoptosis in cumulus cells may compromise the competence of oocyte in diabetic mice through the following possible pathways. First, reduced ATP levels in cumulus cells (Fig. 5) and decreased oocyte-somatic cell gap junction communication may together contribute to the low ATP content we observed in diabetic oocytes [7], [8]. Such a variation in ATP content has been suggested to significantly affect oocyte quality, embryonic development and even the implantation process [60], [61]. Second, potential oxidative stress in the cumulus cells of diabetic mice may create an unfavorable condition for oocyte development. Mitochondria are the major reactive oxygen species (ROS) generator, as well as one of the main target of ROS-induced oxidative damage [62]. By performing DCFDA staining on cumulus-oocyte complexes (see File S1), we found that ROS production is significantly increased in cumulus cells from diabetic mice when compared to those from control mice (Fig. S5A–B), which is probably the result of glucose deprivation and mitochondrial dysfunction [62], [63], [64]. mtDNA is highly susceptible to oxidative attack because of its lack of both protective histones and DNA repair activity [65]. Thus, it is possible that mitochondrial dysfunction give rise to oxidative stress in cumulus cells of diabetic mice, leading to mtDNA damage. Notably, mtDNA deletion in granulosa cells has been proposed as a factor affecting oocyte quality of aged women [66], [67]. Furthermore, oocytes exposed to environmental oxidative stress exhibit the accelerated aging phenomena [68]. Finally, apoptosis of cumulus cells may compromise developmental capability of the oocyte. Gap junction intercellular communication has been demonstrated to be able to spread cell-injuring signals generated by cells undergoing apoptosis into healthy neighbors [69], [70], [71]. Moreover, granulosa cell apoptosis was found to be accelerated in human patients with unexplained infertility [72]. Increased apoptosis of the surrounding cumulus cells has been correlated with oocyte maturation delay and poor pregnancy outcomes [73], [74], [75], [76]. It is therefore possible that toxic metabolites from cumulus cells undergoing apoptosis travels through gap junctions into the oocyte. All together, maternal diabetes may indirectly impair oocyte competence by disrupting mitochondrial function in cumulus cells and their communications with the oocyte.

In conclusion, our results suggest a potential mechanism by which maternal diabetes affects oocyte quality. Together with our previous findings detailing the mitochondrial dysfunction in oocytes from diabetic mice [8], targeting drugs to override conditions that lead to mitochondrial damage and/or improve mitochondrial function may have therapeutic potential in treating reproductive failure of diabetic females.

## Materials and Methods

### Ethics Statement

All mouse studies were approved by the Animal Studies Committee at Washington University School of Medicine and conform to the Guide for the Care and Use of Laboratory Animals published by the National Institutes of Health. Female B6SJLF1 mice (age 20–24 days; Jackson Laboratories, Bar Harbor, ME) were used in this research.

### Generation of diabetic mice

To generate a type I diabetic model, female mice received a single injection of streptozotocin (Sigma, St. Louis, MO) at a dose of 190 mg/kg (dissolved in sodium citrate buffer, pH 4.4). Four days after injection, glucose concentration was measured from tail-blood samples via a Hemocue B glucose analyzer (Stockholm, Sweden). If glucose levels were greater than 300 mg/dl, the animal was selected for use as a diabetic model. Control mice were also randomly selected, and their blood sugar was checked to ensure that it was less than 150 mg/dl.

### Akita mice

Akita mice have an autosomal dominant mutation, resulting in hyperglycemia and notable pancreatic β-cell dysfunction [30]. All Akita mice (FVB.B6-Ins2Akita/MlnJ; The Jackson Laboratory, Stock No: 006867) had glucose checked at approximately 5–7 wk through a tail-blood sample. If glucose levels were more than 250 mg/dl, these mice were considered to have the mutation. Age matched wild type mice were FVB/N females with glucose levels ∼100 mg/dl.

### Cumulus-oocyte complex retrieval

Diabetic mice and age-matched controls were superovulated with 10 IU Pregnant Mares Serum Gonadotropin (PMSG; Sigma) by intraperitoneal injection. 48 hours later, the ovaries were removed and placed in a dish containing M2 medium (Sigma). Cumulus-oocyte complexes (COCs) were obtained by manual rupturing of antral ovarian follicles with a sterile needle. For some experiments, cumulus cells were collected by mouth pipetting the COCs repeatedly.

### Transmission electron microscopy

For ultrastructural analysis of mitochondria, COCs were processed for transmission electron microscopy (TEM) as described previously [77]. Evaluations of mitochondrial morphology were accomplished by taking TEM micrographs from randomly selected areas of cumulus cells. To quantify the abnormal mitochondria, for each group, 15 micrographs taken from 15 COCs from three mice were counted in blinded fashion at 25,000× magnification.

### Estimation of mitochondrial membrane potential (Δψ_m_) in cumulus cells

Mitochondrial membrane potential was assessed using the MitoPT-JC1 assay kit (#924; Immunochemistry, Bloomington, MN). Briefly, COCs were incubated in assay buffer with JC-1 for 15 min at 37°C. After two washes, cells were mounted on slides with a drop of assay buffer and then examined using fluorescence microscope. In healthy cells with high Δψ_m_, JC-1 spontaneously forms complexes known as J-aggregates with intense red fluorescence. In contrast, in unhealthy cells with low Δψ_m_, JC-1 remains in the monomeric form, which shows only green fluorescence [19]. The ratios of red/green fluorescence intensity were calculated to characterize the Δψ_m_ [78], [79]. To quantify the fluorescence intensity, 10 different regions taken from 10 COCs were randomly selected from 10 images for each group. These regions were measured and quantified by Image J (National Institute of Health).

### Immunofluorescence

For mitochondria localization, COCs were cultured in M2 medium containing 200 nM MitoTracker Red (Molecular Probes, Eugene, OR) for 30 min at 37°C. Following washes, COCs were fixed with 4% paraformaldehyde for 20 minutes and then treated with 0.5% Triton X-100 for 20 minutes. After the brief counterstaining with 4′,6′-diamidino-2-phenylindole (DAPI), samples were analyzed by confocal microscopy (Meta 510; Carl Zeiss, Germany).

For cytochrome *c* subcellular distribution, COCs were labeled with MitoTracker Red as described above, and then fixed with 4% paraformaldehyde for 20 minutes and permeabilized with 0.5% Triton X-100 for 20 minutes. Followed by blocking in 1% BSA-supplemented PBS for 1 hour, samples were incubated with mouse monoclonal cytochrome *c* antibody (BD Biosciences, San Jose, CA) overnight at 4°C. In some experiments, samples labeled with cytochrome *c* antibody were also co-labeled with rabbit polyclonal cleaved caspase-3 antibody (Cell Signaling Technology, Danvers, MA). Alexa 488 goat anti-mouse and/or Alexa 594 goat anti-rabbit secondary antibodies were then applied for 1 hour at room temperature as appropriate. Nuclear status was evaluated by DAPI staining. Samples were examined by confocal microscopy (Meta 510; Carl Zeiss, Germany).

### Determination of mtDNA content in cumulus cells

Total DNA was extracted from cumulus cells removed from COCs using a DNeasy kit (QIAGEN, Chatsworth, CA). mtDNA content was calculated using quantitative real-time PCR as described previously [47], [80] by measuring the threshold cycle ratio (ΔCt) of a mitochondria-encoded gene (*cyto b*, forward 5′ CCACTTCATCTTACCATTTATTATCGC, reverse 5′ TTTTATCTGCATCTGAGTTTAATCCTGT) versus a nuclear-encoded gene (*β-actin*, forward 5′ CTGCCTGACGGCCAGG, Reverse 5′ CTATGGCCTCAGGAGTTTTGTC). Data are expressed as mtDNA/nuclear DNA. All measurements were performed in triplicate.

### Real-Time RT-PCR

Cumulus cells removed from 200 COCs per group were collected for total RNA extraction using an RNeasy kit (QIAGEN). 200 ng of RNA was used for cDNA synthesis. Real-time RT-PCR assay was performed according to the detailed procedure described by Ratchford et al. [16]. Each reaction was run in triplicate and consisted of 10 ng cDNA, 1× *Power* SYBR Green PCR System (Applied Biosystem, Foster City, CA) and 4 µM forward/reverse primers. The fold change in gene expression was calculated using the ΔΔCt method [81] with the house keeping gene, glyceraldehydes-3-phosphate dehydrogenase (GAPDH), as the internal control. Primer sequences are listed below:

PGC-1α-for: AAGTGTGGAACTCTCTGGAACTG;

PGC-1α-rev: GGGTTATCTTGGTTGGCTTTATG;

NRF1-for: CTCATCCAGGTTGGTACAGG;

NRF1-rev: GTCGTCTGGATGGTCATTTC;

TFAM-for: AATGTGGAGCGTGCTAAAAG;

TFAM-rev: AGCTGTTCTGTGGAAAATCG;

GAPDH-for: ATTGTCAGCAATGCATCCTG;

GAPDH-rev: ATGGACTGTGGTCATGAGCC.

### Metabolite analytic assays

Cumulus cells removed from 100 COCs were homogenized in 10 µl 0.1N NaOH. The homogenate was heated to 80°C for 20 min, and then 5 µl of 0.15N HCl and 0.1 M Tris-HCl (pH 6.6) cocktail was added to maintain a pH of 8.1. The samples were stored at −80°C until the analytic assays were performed to determine the ATP and citrate levels. Assays were designed to link reactions ending with NAD/NADH or NADP/NADPH, which then were enzymatically amplified in a cycling reaction, and a byproduct of the amplification step was measured in a fluorometric assay. The detailed assay conditions are described in Chi et al. [82]. Reactions are normalized to total DNA, and metabolite contents are expressed as pmol/µg DNA.

### Terminal dUTP nick end labeling (TUNEL) assay

Cumulus cells apoptosis was evaluated using TUNEL (Roche Molecular Biochemicals, German) as previously described [83]. COCs were fixed in 4% paraformaldehyde in PBS, permeabilized with 0.1% Triton X-100, and then incubated in fluorescence-labeled dUTP and terminal transferase for 1 hour at 37°C in the dark. Nuclear DNA was counterstained with DAPI. Complexes were visualized using confocal microscopy (Meta 510; Carl Zeiss, Germany). Apoptosis was expressed as the percentage of TUNEL-positive nuclei per COC. These experiments were performed in triplicate with 10 COCs per group for each experiment.

### Statistical analysis

Data are presented as mean ± SD, unless otherwise indicated. Statistical comparisons were made with Student's *t* test and ANOVA when appropriate. P< 0.05 was considered to be significant.

## Supporting Information

Figure S1 **GLUT1 protein expression is downregulated in cumulus cells of diabetic mice.** Cumulus cells isolated from cumulus-oocyte complexes were processed for Western blot to analyze GLUT1 protein expression, and β-actin was used as an internal control for loading variability. Representative Western blots showing the decreased GLUT1 expression in cumulus cells from (A) Streptozotocin (STZ)-induced diabetic and (B) Akita mice compared to their controls.
(TIF)

Figure S2 **Decreased glucose uptake in cumulus cells of diabetic mice.** Glucose uptake was measured in cumulus cells from (A) control/STZ-induced diabetic and (B) WT/Akita mice, respectively, and each sample was normalized to total protein. Error bars indicate ± SD. * p<0.05.
(TIF)

Figure S3 **Reduced mitochondrial membrane potential in cumulus cells of Akita mice.** Cumulus-oocyte complexes (COCs) from wild type and Akita mice were stained with JC-1 to evaluate mitochondrial membrane potential (Δψ_m_) by fluorescence microscopy. Representative images are shown. (A) Mitochondria in WT cumulus cells were predominantly in red form, indicating the high Δψ_m_. The loss of red fluorescence and increased green mitochondria were observed in Akita cumulus cells. (B) Histogram shows the ratio of red to green fluorescence intensity calculated to characterize Δψ_m_. Note the decreased Δψ_m_ in Akita cumulus cells. Error bars indicate ± SD. * p<0.05. Scale bar: 20 µm.
(TIF)

Figure S4 **Increased apoptosis in cumulus cells of Akita mice.** (A) Cumulus-oocyte complexes from wild type and Akita mice were stained with TUNEL to visualize apoptotic cells (red) and counterstained with DAPI to visualize nuclei (blue). Representative confocal sections of cumulus cells are shown. (B) Frequency of TUNEL-positive nuclei in cumulus cells from WT and Akita mice. Data represent mean ± SD of three independent experiments in which at least 30 COCs were analyzed. * p<0.05.
(TIF)

Figure S5 **Increased ROS production in cumulus cells of diabetic mice.** (A) Cumulus-oocyte complexes (COCs) from control and STZ-induced diabetic mice were stained with DCFDA to determine ROS production by fluorescence microscopy. Representative images are shown. (B) Histogram shows the increased fluorescence intensity in cumulus cells of diabetic mice. Error bars indicate ± SD. * p<0.05. Scale bar: 20 µm.
(TIF)

File S1 **Additional materials and methods.**
(DOC)
