# Supplementary figures and images for: Mitochondrial Dysfunction and Apoptosis in Cumulus Cells of Type I Diabetic Mice
Source: PLoS One. 2010 Dec 28;5(12):e15901. doi: 10.1371/journal.pone.0015901 (PMC3011018; doi:10.1371/journal.pone.0015901)

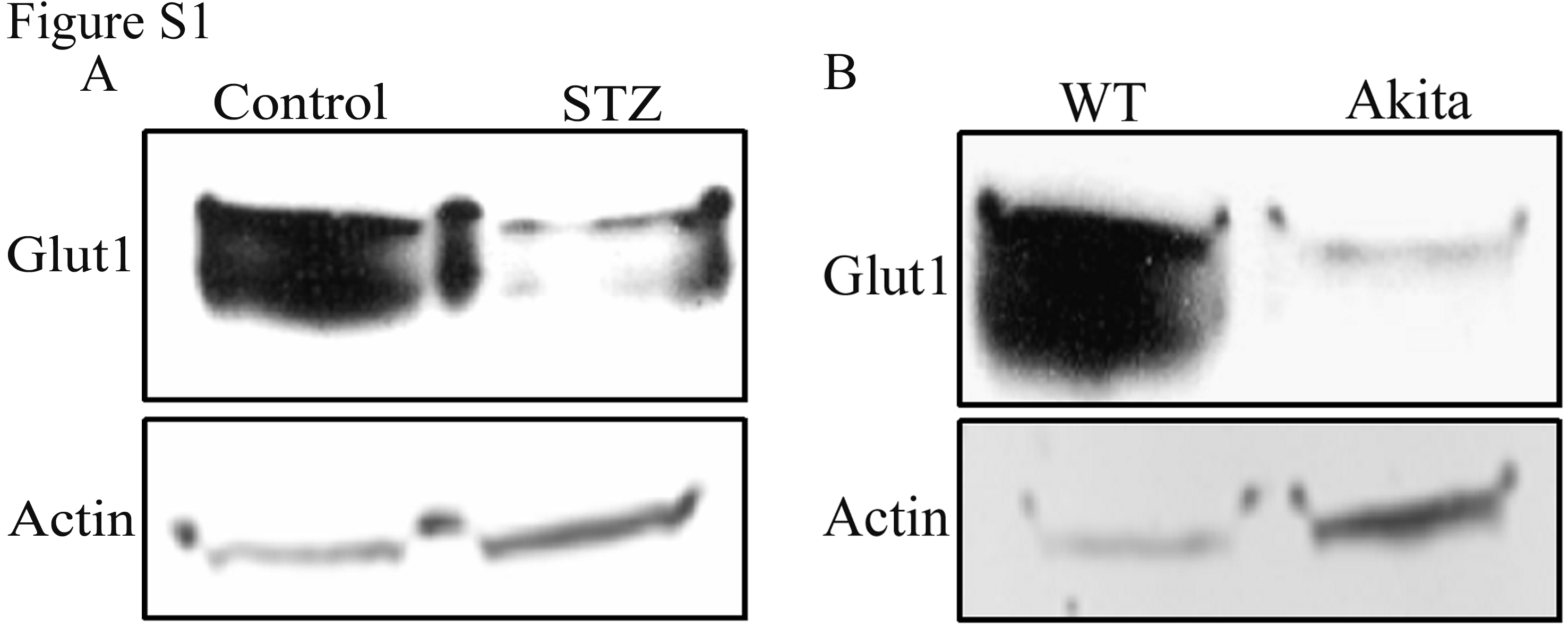

Supplement: Figure S1 — GLUT1 protein expression is downregulated in cumulus cells of diabetic mice. Cumulus cells isolated from cumulus-oocyte complexes were processed for Western blot to analyze GLUT1 protein expression, and β-actin was used as an internal control for loading variability. Representative Western blots showing the decreased GLUT1 expression in cumulus cells from (A) Streptozotocin (STZ)-induced diabetic and (B) Akita mice compared to their controls. (TIF) [file pone.0015901.s001.tif]

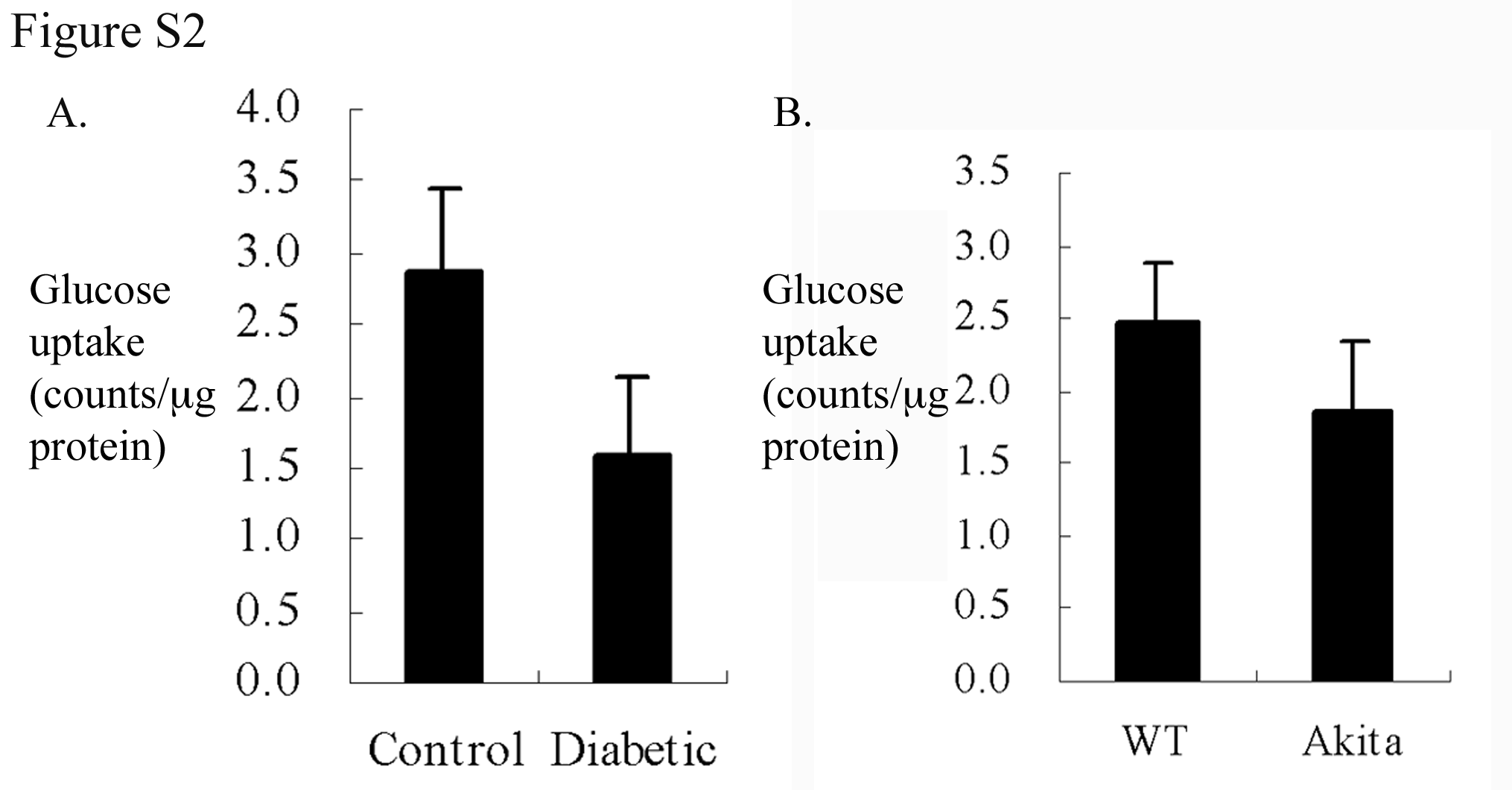

Supplement: Figure S2 — Decreased glucose uptake in cumulus cells of diabetic mice. Glucose uptake was measured in cumulus cells from (A) control/STZ-induced diabetic and (B) WT/Akita mice, respectively, and each sample was normalized to total protein. Error bars indicate ± SD. * p<0.05. (TIF) [file pone.0015901.s002.tif]

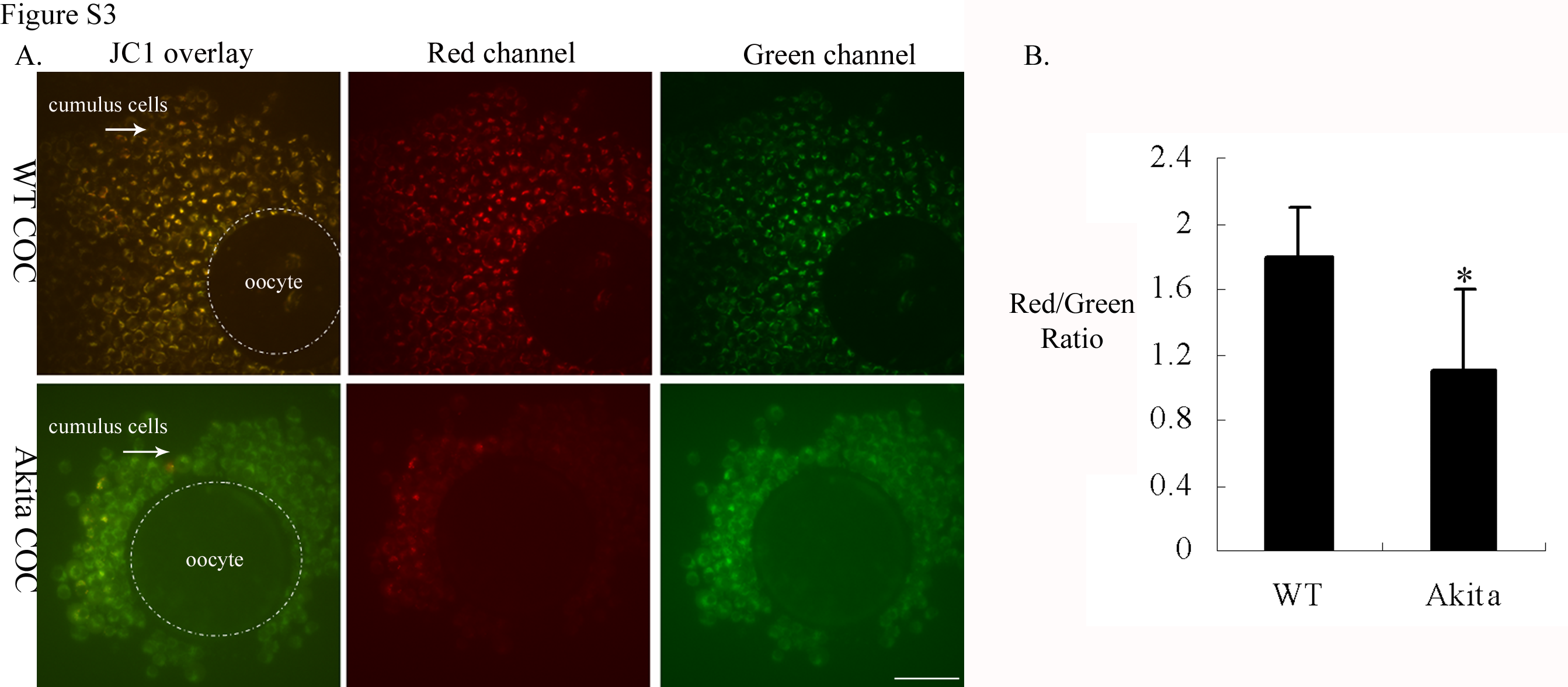

Supplement: Figure S3 — Reduced mitochondrial membrane potential in cumulus cells of Akita mice. Cumulus-oocyte complexes (COCs) from wild type and Akita mice were stained with JC-1 to evaluate mitochondrial membrane potential (Δψm) by fluorescence microscopy. Representative images are shown. (A) Mitochondria in WT cumulus cells were predominantly in red form, indicating the high Δψm. The loss of red fluorescence and increased green mitochondria were observed in Akita cumulus cells. (B) Histogram shows the ratio of red to green fluorescence intensity calculated to characterize Δψm. Note the decreased Δψm in Akita cumulus cells. Error bars indicate ± SD. * p<0.05. Scale bar: 20 µm. (TIF) [file pone.0015901.s003.tif]

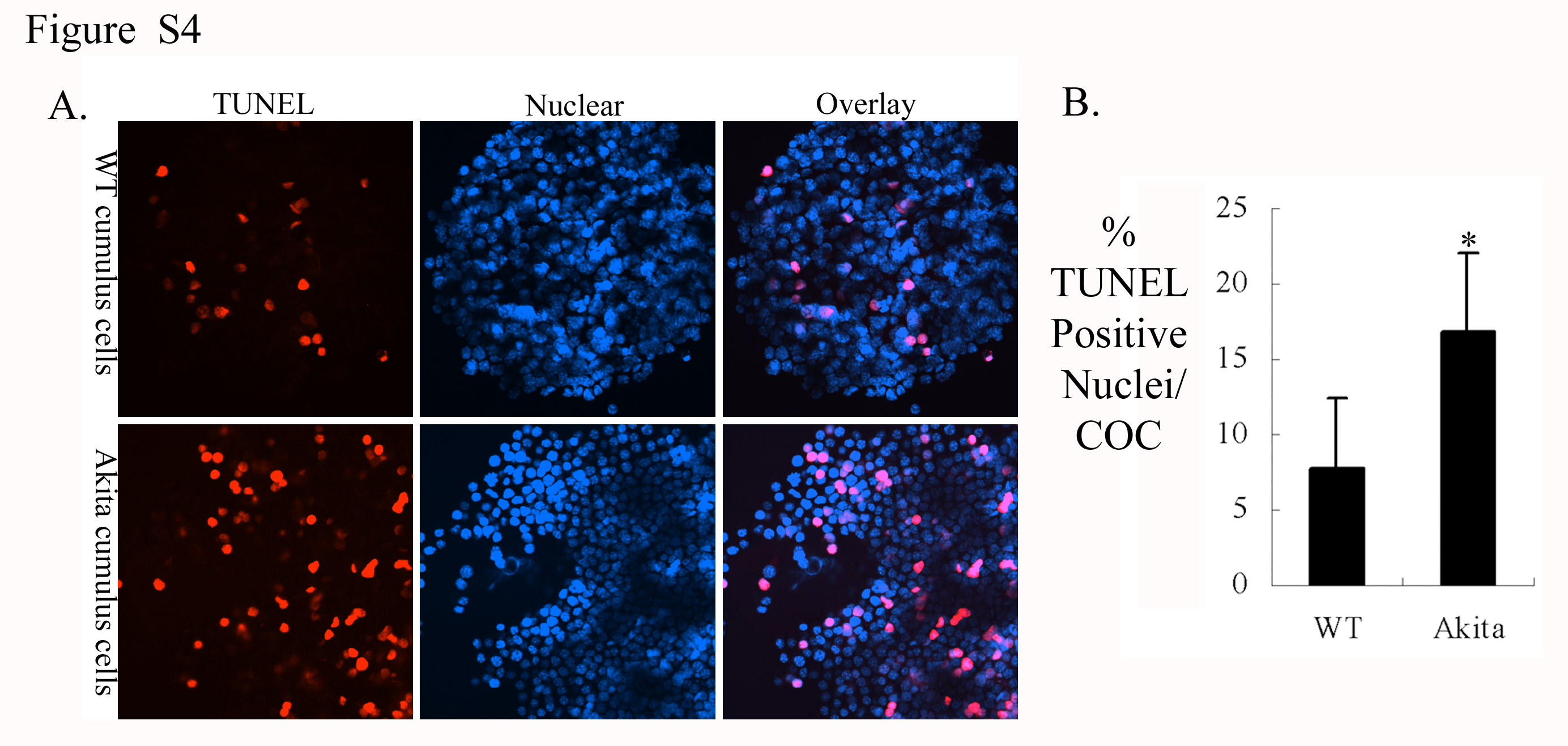

Supplement: Figure S4 — Increased apoptosis in cumulus cells of Akita mice. (A) Cumulus-oocyte complexes from wild type and Akita mice were stained with TUNEL to visualize apoptotic cells (red) and counterstained with DAPI to visualize nuclei (blue). Representative confocal sections of cumulus cells are shown. (B) Frequency of TUNEL-positive nuclei in cumulus cells from WT and Akita mice. Data represent mean ± SD of three independent experiments in which at least 30 COCs were analyzed. * p<0.05. (TIF) [file pone.0015901.s004.tif]

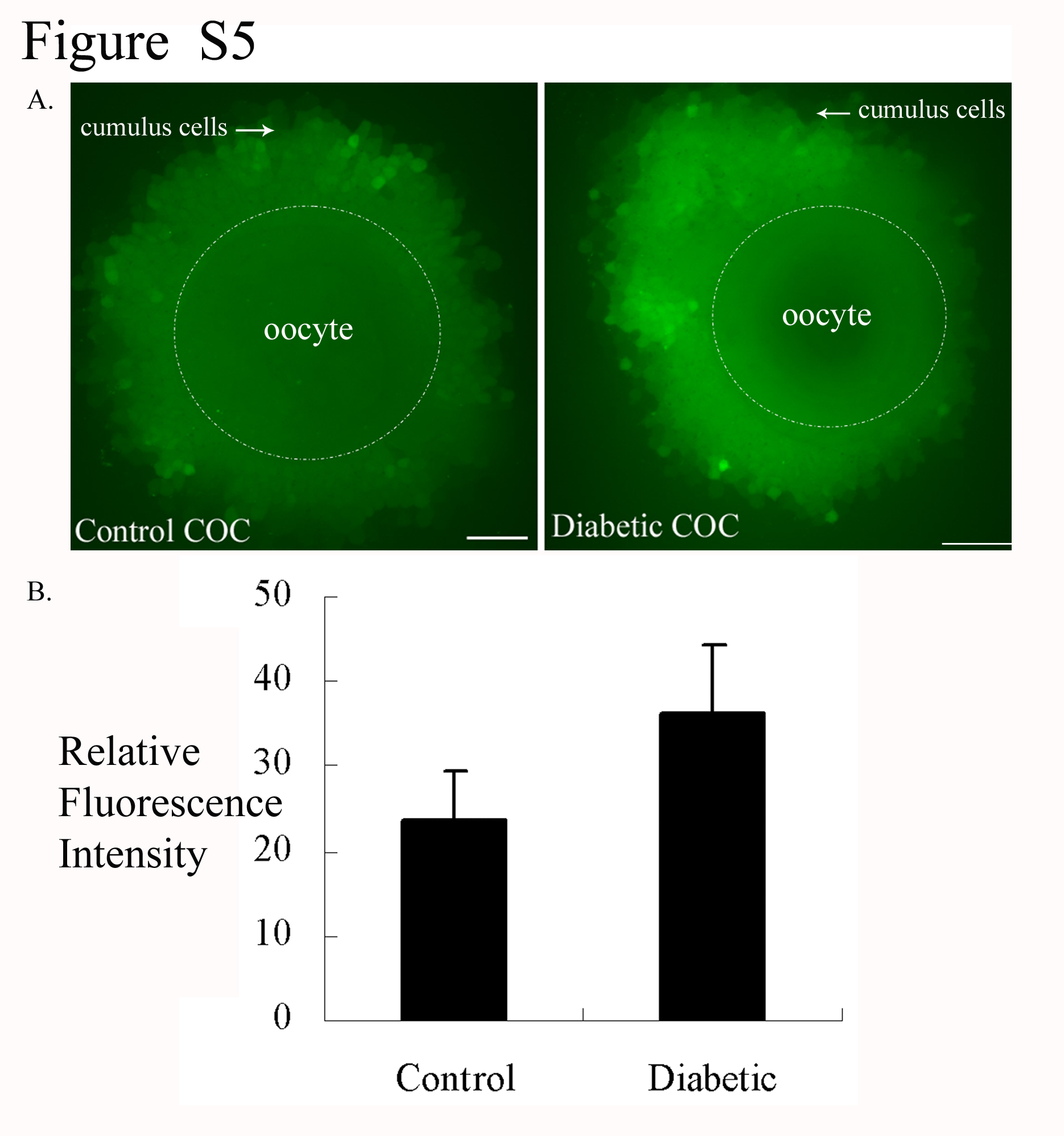

Supplement: Figure S5 — Increased ROS production in cumulus cells of diabetic mice. (A) Cumulus-oocyte complexes (COCs) from control and STZ-induced diabetic mice were stained with DCFDA to determine ROS production by fluorescence microscopy. Representative images are shown. (B) Histogram shows the increased fluorescence intensity in cumulus cells of diabetic mice. Error bars indicate ± SD. * p<0.05. Scale bar: 20 µm. (TIF) [file pone.0015901.s005.tif]
