# Supplementary material for: Mitochondrial Dysfunction and Apoptosis in Cumulus Cells of Type I Diabetic Mice
Source: PLoS One. 2010 Dec 28;5(12):e15901. doi: 10.1371/journal.pone.0015901 (PMC3011018; doi:10.1371/journal.pone.0015901)
Supplement: File S1 — Additional materials and methods. (DOC) [file pone.0015901.s006.doc]

**Supporting Information: File S1**

**Materials and Methods**

**Western blot analysis**

Cumulus cells removed from immature cumulus-oocyte complexes were homogenized in 50 µl lysis buffer containing 50 mM Tris HCl (pH 7.5) 150 mM NaCl, 5 mM EDTA, 0.5% NP-40, and complete protease inhibitor cocktail (Roche Applied Science, Indianapolis, IN). The total proteins were loaded and separated using SDS PAGE and transferred to nitrocellulose. Blots were blocked in Tris-buffered saline containing 0.05% Tween 20 and 5% nonfat dry milk for 1 hour and then incubated with rabbit anti-GLUT1 (1:3000) kindly provided by Dr. Michael Mueckler, Washington University and mouse anti-β-actin (1:5000; Millipore, Billerica, MA) overnight at 4^o^C. Secondary goat anti-rabbit IR Dye 800 or goat anti-mouse IR Dye 680 antibodies (LI-COR Biosciences, Lincoln, NE) were incubated for 1 hour at room temperature at 1:10,000 dilution. Membranes were scanned using the Odyssey fluorescent imager (LI-COR Biosciences). Experiments were replicated three times and confirmed in the Akita model as well.

**Glucose uptake assay**

Cumulus cells removed from immature cumulus-oocyte complexes were washed in PBS and then incubated in 50 µl of Krebs-Ringer buffer without glucose for 10 min at 37 ^o^C. At the end of incubation, cells were washed with PBS and incubated in uptake buffer containing ^3^H-2-deoxyglucose for 1 min. The glucose uptake was stopped by incubating the cells in 10 mM cytochalasin B for 1 min. Following two washes, cells were divided into two groups to measure protein concentrations using BCA assay and to determine ^3^H radioactivity on scintillation counter, respectively. Reactions are normalized to total protein, and values are expressed as counts/µg protein.

**Evaluation of reactive oxygen species (ROS) production**

ROS production was measured by [2',7'-](http://www.anaspec.com/products/product.asp?id=29594)dichlorodihydrofluorescein diacetate (DCFDA; Sigma, MO). In brief, cumulus-oocyte complexes (COCs) were incubated in culture medium with 10 µm DCFDA for 15 min at 37 ^o^C. After two washes, cells were loaded on slides with a drop of mounting medium and then examined using fluorescence microscope. To quantify the fluorescence intensity, for each group, 10 different regions randomly selected from 10 images taken from 10 COCs were measured by Image J.
